# Aloe-emodin attenuates hyperuricemia-induced renal injury in mice by mitigating inflammation and oxidative stress

**DOI:** 10.3389/fnut.2025.1677560

**Published:** 2025-10-15

**Authors:** Shengfeng Wang, Quanfeng Zhu, Chengcheng Zhang, Shuang Hu, Daqun Liu, Jiawen Yu, Xiao Liu, Yan Chen, Guojun Jiang

**Affiliations:** ^1^Laboratory of Animal Center, Research Center of Analysis and Measurement, Zhejiang University of Technology, Hangzhou, China; ^2^Affiliated Xiaoshan Hospital, Hangzhou Normal University, Hangzhou, China; ^3^Food Science Institute, Zhejiang Academy of Agricultural Sciences, Hangzhou, China; ^4^School of Pharmacy, Hangzhou Normal University, Hangzhou, China

**Keywords:** aloe-emodin, hyperuricemia, renal injury, oxidative stress, inflammatory response, PPAR/NF-κB signaling

## Abstract

**Background:**

Aloe-emodin (AOE), the principal anthraquinone constituent derived from aloe and rhubarb, exhibits antioxidant and anti-inflammatory properties, suggesting its therapeutic potential against hyperuricemia (HUA) and associated renal injury. Here, we investigated the potential of AOE in mitigating HUA and related kidney damage, with a focus on its underlying biological mechanisms.

**Methods:**

A HUA mouse model was established by oral gavage of potassium oxonate (PO, 1.5 g/kg) and adenine (Ad, 0.1 g/kg). Serum uric acid (UA) levels, kidney function indicators, histological changes, inflammatory response, and oxidative stress state were assessed to evaluate the urate-lowering and kidney-protective roles of AOE. Furthermore, transcriptomic profiling and RT-qPCR analysis were employed to investigate how AOE contributes to UA reduction and renal protection.

**Results:**

AOE lowered serum UA levels and inhibited xanthine oxidase and adenosine deaminase activity. Moreover, AOE improved kidney function indicators (reflected by reductions in serum creatinine and blood urea nitrogen levels), restored the integrity of renal tissue structure, and mitigated inflammation and oxidative stress in HUA-exposed animals. Transcriptomic analysis revealed 2,307 differentially expressed key genes associated with AOE against HUA in kidney. Furthermore, AOE downregulated p65/RelA and NF-κB1/p50 transcript levels, while increasing *PPARα*, *PPARγ*, and *CPT2* expression.

**Conclusion:**

AOE effectively lowered serum UA levels, and exhibited renal protection in the PO/Ad-induced HUA mouse model by dampening inflammatory signaling and restoring redox equilibrium, likely through the PPAR and NF-κB pathways. This study demonstrated that AOE is a promising natural candidate with a desirable safety profile for treating HUA and renal injury, and more experimental validation are needed in the future.

## 1 Introduction

Hyperuricemia (HUA) is a metabolic disorder caused by either purine metabolism dysfunction or insufficient uric acid clearance. It is the world’s second most common metabolic condition after diabetes (1, 2). Among Chinese adults, reported rates of HUA are 24.4% in males and 3.6% in females (3), with an increasing prevalence among younger individuals (4). Over the past few decades, with changes in lifestyle and dietary patterns, including the consumption of purine-rich and protein-heavy diets, HUA and its complications, such as gout, hyperuricemic nephropathy (HN), and renal failure, have been increasing rapidly (3, 5). Moreover, HUA is associated with the development of multiple target organ damage (6), with HN being the most common consequence, as the kidney is the primary organ for the excretion and reabsorption of UA (7, 8). Emerging evidence suggests that HUA directly induces renal pathologies, including acute and chronic kidney impairments, and may also promote advancement to end-stage renal disease (9, 10). However, effective strategies for HUA management remain missing. Currently, HUA treatment focuses on suppressing the generation of UA (febuxostat, allopurinol) and promoting the excretion of UA (benzbromarone) (9, 11). However, uric acid-lowering drugs may exacerbate hepatic and renal damage because of side effects like hepatic/renal dysfunction and gastrointestinal adverse reactions. This highlights the need to develop treatments with improved safety and efficacy for HUA and its associated renal complications.

HUA-induced renal damage is frequently accompanied by inflammatory responses (12, 13). Chronic exposure to elevated UA levels can initiate the activation of the nuclear factor-kappa B (NF-κB) inflammatory signaling pathway, promoting the production of pro-inflammatory cytokines and leading to an inflammatory response (12). Notably, activation of NF-κB has been observed in the renal proximal tubule cells of HUA mice (14), and it can be modulated by a diverse array of cellular signaling cascades, including those involving peroxisome proliferator-activated receptors (PPARs), mitogen-activated protein kinases (MAPK), and reactive oxygen species (ROS) (15). Previous studies have shown that PPARγ exhibits anti-inflammatory properties by inhibiting the NF-κB signaling cascade and inflammatory mediators (16). These studies suggest that the PPAR and NF-κB pathways may be potential targets for mitigating renal inflammation and present a promising novel therapy for HUA-induced renal damage.

Phytochemicals isolated from Chinese medicinal and edible plants show compelling therapeutic effects against HUA, with fewer toxic and adverse reactions (9, 17). Notably, previous evidences have indicated that anthraquinone components possess the ability to reduce UA levels and alleviate inflammation (18). For instance, rhubarb acid, the main anthraquinone compound in *Rheum palmatum*, can decrease serum UA levels in adenine and ethambutol-induced HUA mouse models, and improve HUA-induced renal damage by suppressing inflammatory mediators (19). Moreover, emodin, another natural anthraquinone, also shows promise in the treatment of HUA and its associated gout, primarily by increasing renal uric acid excretion (20). Aloe-emodin (AOE) is an anthraquinone bioactive component, which is primarily sourced from *Aloe vera*, *Rheum officinale*, and *Cassiae semen* (21). AOE possesses extensive biological activities, including antioxidant (22), anticancer (23), and anti-inflammatory properties (24). Regarding its hypouricemic activity, AOE exhibits a strong inhibitory effect on xanthine oxidase (XOD) (25). XOD and adenosine deaminase (ADA) are critical purine degradation enzymes that convert hypoxanthine to xanthine, promoting uric acid production (26). Considering the potential beneficial effects of AOE, we hypothesize that it is a strong natural candidate for the therapeutic management of HUA and its related kidney complications. Nonetheless, to date, the therapeutic efficacy of AOE against HUA and HUA-induced kidney injury remains unclear, and its underlying mechanisms have not been thoroughly investigated or reported.

Our study aimed to assess the efficacy of AOE in treating HUA and associated renal impairment. To determine its mode of action, we conducted extensive transcriptome profiling and real-time quantitative reverse transcription PCR (RT-qPCR) on renal tissue. The outcomes of this investigation may help advance AOE as a therapeutic option for hyperuricemia and its related renal complications.

## 2 Materials and methods

### 2.1 Chemicals and reagents

Aloe-emodin (AOE, B20772) was obtained from Yuanye Bio-Technology Co., Ltd. (Shanghai, China). Information regarding additional substances used, such as reagents, assay kits, and primers, is available in the Supplementary materials.

### 2.2 Animal experimental methods

All procedures involving animals were conducted in accordance with approval from the Animal Ethics Committee of Zhejiang University of Technology (Ethics No. ZH20250305040). Male ICR mice were purchased from Shanghai SLAC Laboratory Animal Co., Ltd. (Shanghai, China).

**Figure 1:**
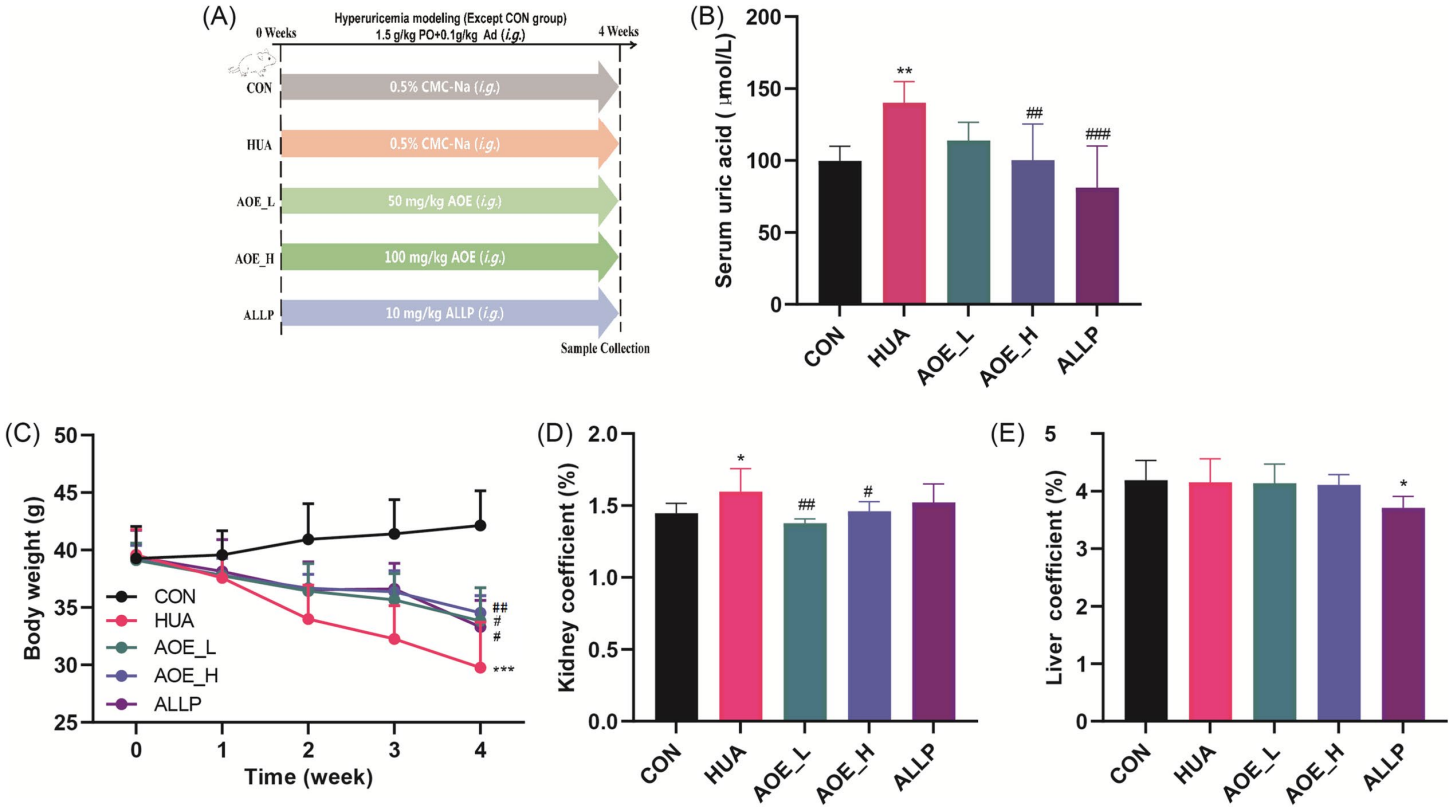
Influence of AOE on serum uric acid level, body weight, and organ coefficients in HUA mice. **(A)** Schematic diagram depicting the experimental protocol in HUA mice; **(B)** Serum uric acid level; **(C)** Body weight changes; **(D)** Kidney and **(E)** Liver coefficient. Values are expressed as mean ± standard deviation (SD). ^∗^*p* < 0.05, ^∗∗^*p* < 0.01, ^∗∗∗^*p* < 0.001 *vs* the CON group, and ^#^*p* < 0.05, ^##^*p* < 0.01, ^###^*p* < 0.001 *vs* the HUA group.

Following a one-week of acclimatization, mice were randomly divided into five groups (*n* = 10 per group; Figure 1A): a control group (CON, receiving 0.5% CMC-Na), a hyperuricemia model group (HUA, also receiving 0.5% CMC-Na), a low-dose AOE group (AOE_L, 50 mg/kg), a high-dose AOE group (AOE_H, 100 mg/kg), and a positive control group (ALLP, 10 mg/kg). To induce hyperuricemia, all groups except the control were administered adenine (0.1 g/kg, p.o.) and potassium oxonate (1.5 g/kg, p.o.) suspended in 0.5% CMC-Na daily for 4 weeks (27). The PO/Ad-induced mouse model has been widely used for HUA establishment. The mice were then administered the corresponding drugs intragastrically after 3 h of Ad and PO treatment. The selection of AOE dosage was based on our preliminary experiment. Mice body weights were monitored once weekly. After 4 weeks of administration, the animals were fasted for 12 h, and then anesthetized via intraperitoneal injection of sodium pentobarbital at a dose of 50 mg/kg. Blood was collected from orbital venous plexus of mice, followed by euthanasia via cervical dislocation. Blood was centrifuged at 3500 rpm for 10 min at 4 °C to isolate serum, and organs, including the liver and kidneys, were excised and weighed. Serum, liver, and left kidney samples were frozen at −80 °C; the right kidney was formalin-fixed and stained with hematoxylin and eosin (H&E) for histology.

### 2.3 Measurement of serum biochemical indices

Levels of serum UA, creatinine (Cr), and blood urea nitrogen (BUN) were quantified using an automatic biochemistry analyzer (Chemray 240, Rayto, China).

### 2.4 Assessment of XOD and ADA enzymatic activity

Liver samples were processed by blending one part of tissue with nine parts of physiological saline to produce a 10% (w/v) homogenate using a high-speed homogenizer. The resulting solution was centrifuged at 3,000 rpm for 15 min, and the supernatant collected. Enzymatic activities of XOD and ADA in liver homogenates and serum were quantified using commercially available kits, strictly following the manufacturer’s protocols.

### 2.5 Determination of inflammatory factors

Pro-inflammatory cytokine (IL-6, IL-1β, and TNF-*α*) concentrations in serum and kidney homogenates were determined via enzyme-linked immunosorbent assay (ELISA) according to the manufacturer’s recommended procedures. Cytokine levels in kidney samples were normalized against the total protein content of the corresponding homogenates.

### 2.6 Determination of oxidative stress levels

Kidney tissues were homogenized in PBS and centrifuged. The supernatant was then assessed for oxidative stress markers. Levels of MDA and the enzymatic activities of SOD, GSH-Px, and CAT in serum and kidney homogenates were measured using commercial assay kits.

### 2.7 Kidney histology

Kidney specimens were fixed in 10% neutral-buffered formalin for 24 h, then subjected to standard paraffin embedding and H&E staining for histopathological examination. Tissue sections were analyzed under a light microscope to assess morphological changes and the degree of renal injury.

### 2.8 Molecular docking

Binding interactions between AOE and the target enzymes XOD and ADA were investigated through molecular docking simulations conducted using AutoDock Vina (28). The crystal structures of XOD (PDB ID 2HD1) and ADA (PDB ID 3IAR) were obtained from the Protein Data Bank.^1^ Then, the pretreatment of enzyme molecules was performed, which includes cleaning, correcting, removing ligands and water, and adding hydrogen, using PyMOL software (version 3.0.3). The docking pocket of XOD was defined with coordinates center_x = −4.1, center_y = 15.6, center_z = −18.1, and ADA was defined with coordinates center_x = −3.1, center_y = 0.3, center_z = 0.3. Meanwhile, the docking grids were set to dimensions of 90 Å × 90 Å × 90 Å, with a spacing of 0.375. Subsequently, a genetic algorithm facilitated conformational exploration and evaluation, with docking scores guiding the selection of the most favorable structure for binding mode visualization in PyMOL.

### 2.9 Transcriptome analysis

Total RNA was isolated and purified using TRIzol reagent following the manufacturer’s procedure, and the quantity and purity of total RNA were analyzed using NanoDrop ND-1000 (NanoDrop, Wilmington, DE, USA), followed by electrophoresis with denaturing agarose gel to evaluate the RNA integrity. Library construction for transcriptome sequencing was prepared by using TruSeq Stranded mRNA Library Prep Kit. Then, 2 × 150 bp paired-end sequencing (PE150) were performed on an illumina Novaseq™ 6,000 at LC-Bio Technologies (Hangzhou) Co., Ltd. following the vendor’s recommended protocol.

The initial sequencing data underwent quality control to identify high-confidence gene transcripts, reads those contained adapters, those with >20% low-quality bases, those containing poly A and G, and those undetermined bases were removed with fastp software^2^. The expression levels of transcripts were then quantified using Feature Counts.^3^ To identify differentially expressed genes (DEGs) between groups, RSEM^4^ was used to generate transcript per million (TPM) values at the gene level, and DESeq2 was applied to screen for DEGs with log_2_FC > 1.5, *p* < 0.05, and false discovery rate (FDR) < 0.05. Subsequently, we conducted functional annotation and pathway enrichment of DEGs using Gene Ontology (GO)^5^ and Kyoto Encyclopedia of Genes and Genomes (KEGG), applying hypergeometric distribution methods for analysis of the DEGs. Moreover, correlation analysis, principal coordinate analysis (PCoA), and hierarchical clustering heatmaps were generated using tools available on OmicStudio.^6^

### 2.10 RT-qPCR validation of DEGs

RNA was extracted from kidney tissues, and cDNA was synthesized using a high-capacity cDNA reverse transcription kit (Thermo Fisher Scientific). Subsequently, gene expression levels were quantified utilizing a SYBR Green-based real-time PCR platform (Invitrogen). Primer sequences were custom-synthesized by Shanghai Sangon Biotech and are provided in Supplementary Table S1. Relative quantification of gene expression was calculated with the ΔΔCt method; GAPDH was the internal control.

### 2.11 Statistical analysis

All statistical evaluations were conducted using GraphPad Prism (version 9, Inc., La Jolla, CA, USA). One-way ANOVA followed by Dunnett’s test was applied for group-level comparisons. Data are presented as mean ± SEM, and statistical significance was defined as *p* < 0.05.

## 3 Results

### 3.1 AOE lowers serum urate levels in hyperuricemic mice

To evaluate the urate-lowering potential of AOE, we established a hyperuricemia mouse model by administering potassium oxonate (PO) and adenine (Ad) orally daily for 4 weeks (Figure 1A). Concurrently, AOE was given orally at doses of 50 or 100 mg/kg to assess its therapeutic efficacy in this model. PO/Ad administration significantly increased mice UA serum levels relative to the control group (99.76 vs. 140.15 μmol/L, *p* < 0.01) (Figure 1B). HUA mice treated with high doses of AOE (100 mg/kg) and allopurinol (10 mg/kg) mitigated hyperuricemia in mice (*p* < 0.01), as evidenced by reductions in serum UA levels of 28.4 and 42.1% in the AOE_H and ALLP groups, respectively.

### 3.2 AOE reverses alterations in body weight and organ indices in HUA mice

Body weight and organ-to-body weight ratios were measured to evaluate systemic health and organ impairment in the HUA model (29). Compared to control animals, mice in the hyperuricemia group exhibited a substantial decrease in body weight (*p* < 0.001, Figure 1C) and a marked increase in the kidney coefficient (*p* < 0.05, Figure 1D). AOE intervention prevented weight loss in HUA mice (*p* < 0.05) (Figure 1C). By contrast, the kidney coefficient in the AOE_L and AOE_H groups did not differ from control animals, suggesting that AOE effectively reduced kidney enlargement. Moreover, liver coefficient measurements showed no difference between the HUA, AOE_L, AOE_H, and control groups (Figure 1E). Interestingly, treatment with allopurinol led to a statistically significant decline in the liver coefficient versus controls (*p* < 0.05), suggesting that allopurinol may contribute to hepatic injury.

### 3.3 AOE improves purine metabolism in HUA mice

**Figure 2:**
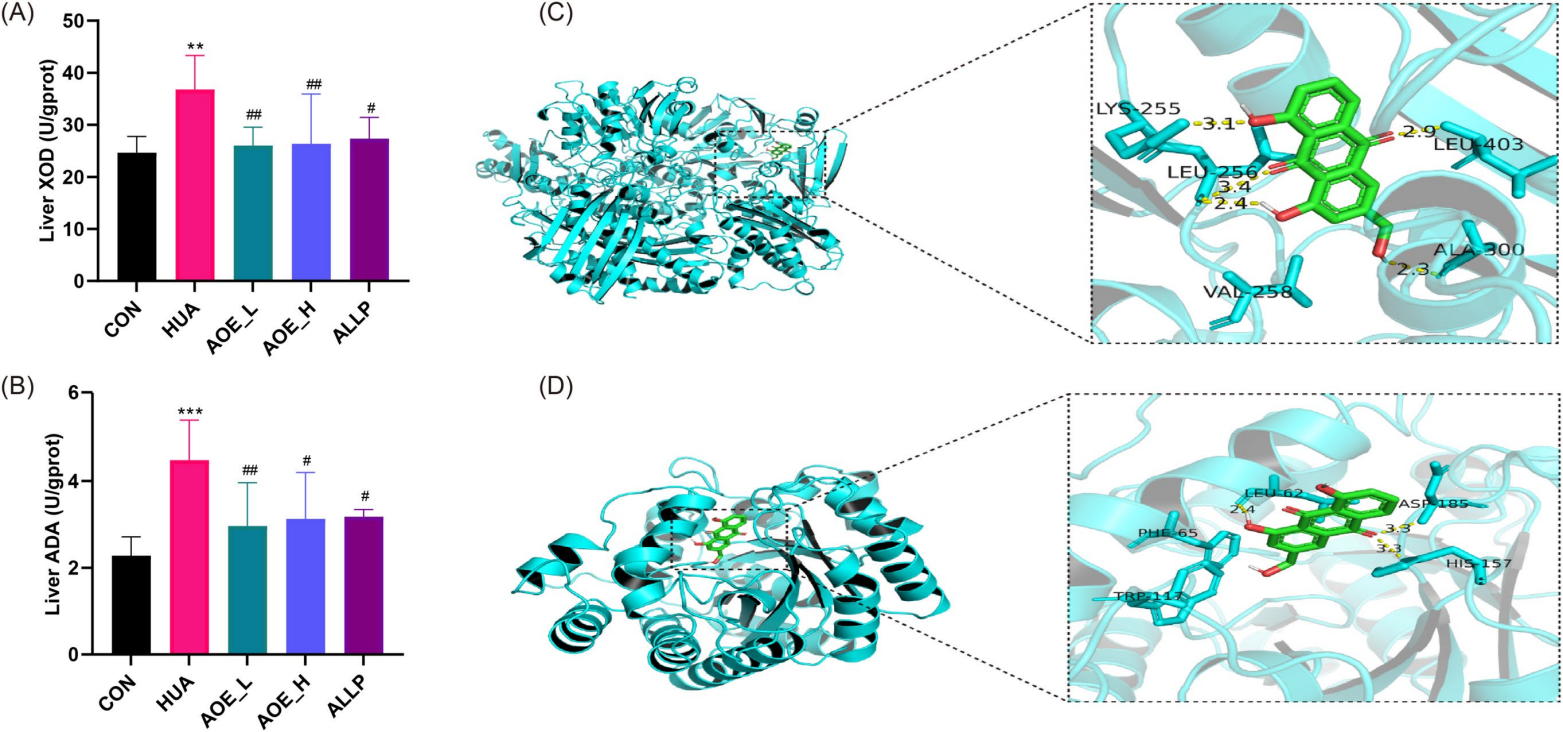
Improvement of purine metabolism in HUA mice by AOE treatment. **(A)** Liver XOD activity; **(B)** Liver ADA activity; **(C)** Docking results of AOE and XOD; **(D)** Docking results of AOE and ADA. Values are expressed as mean ± standard deviation (SD). ^∗^*p* < 0.05, ^∗∗^*p* < 0.01, ^∗∗∗^*p* < 0.001 *vs* the CON group, and ^#^*p* < 0.05, ^##^*p* < 0.01, ^###^*p* < 0.001 *vs* the HUA group.

Hepatic ADA and XOD are crucial enzymes involved in purine catabolism, contributing to the formation of UA (30). Liver samples from HUA mice exhibited significantly elevated activities of XOD and ADA versus control animals (*p* < 0.01) (Figures 2A,B). Treatment with AOE (low- and high doses) (AOE_L and AOE_H) markedly reduced XOD and ADA enzyme activity (*p* < 0.05), indicating that the urate-lowering effect of AOE may be partially attributed to inhibition of ADA and XOD.

To evaluate the binding interactions between AOE and the target enzymes XOD and ADA, we performed molecular docking analysis. In general, a lower affinity value between a ligand and receptor indicates a stronger binding affinity, with binding energy below −6.0 kcal/mol indicating the formation of a stable ligand-receptor complex (31). In this study, the binding energies were calculated to be −9.2 kcal/mol for XOD and −7.3 kcal/mol for ADA, indicating a strong affinity of AOE for both proteins. Docking simulations revealed that AOE forms hydrogen bonds with Lys255, Leu256, Ala300, and Leu403 residues of XOD, and with Leu62, His157, and Asp185 residues of ADA (Figures 2C,D).

### 3.4 AOE ameliorates renal injury in HUA mice

**Figure 3:**
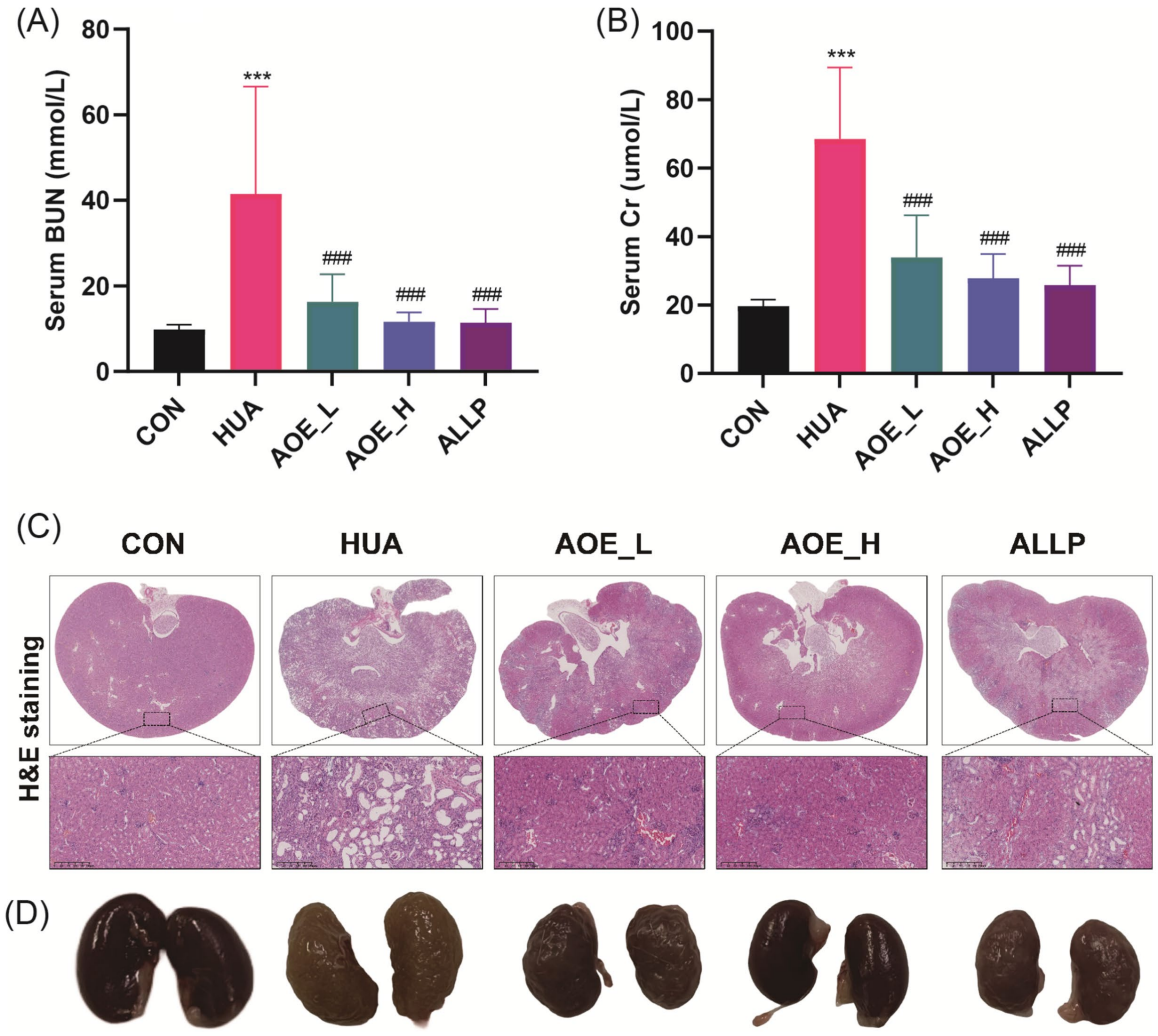
Improvement of kidney injury in HUA mice by AOE treatment. **(A)** Serum creatinine level; **(B)** Blood urea nitrogen level; **(C)** Kidney tissues morphology assessed by hematoxylin and eosin (H&E) staining; **(D)** Digital images of the renal photographs. Values are expressed as mean ± standard deviation (SD). ^∗^*p* < 0.05, ^∗∗^*p* < 0.01, ^∗∗∗^*p* < 0.001 *vs* the CON group, and ^#^*p* < 0.05, ^##^*p* < 0.01, ^###^*p* < 0.001 *vs* the HUA group.

To evaluate renal function in HUA mice, we measured BUN and Cr levels in serum. The HUA group showed significant increases in BUN (41.50 vs. 9.79 mmol/L) and Cr (68.48 vs. 19.69 μmol/L) compared to controls, confirming kidney impairment (Figures 3A,B). AOE-exposure produced a dose-dependent decrease in serum BUN and Cr levels (*p* < 0.001), indicating renal protective effects.

Histopathological analysis revealed distinct alterations in kidney morphology following long-term PO/Ad exposure (13). To evaluate the influence of AOE on renal histopathology in HUA mice, kidney tissue sections were examined microscopically. In the control group, renal cells appeared well-organized with preserved structural integrity, showing no tubular vacuolar dilatation, inflammatory cell infiltration, or glomerular degeneration (Figure 3C). By contrast, the HUA group exhibited glomerular atrophy, marked renal tubular lumen dilatation, and pronounced inflammatory infiltration. These outcomes were not significantly improved by allopurinol treatment; however, AOE intervention restored renal tissue structure and reduced pathological features. Administration of AOE at 100 mg/kg AOE (AOE_H) produced the most significant improvement in kidney damage, with tissue anatomy comparable to control animals. Consistent with the results of H&E staining, kidneys from HUA mice exhibited a pale coloration, noticeable swelling, coarse texture, and were covered with prominent white granules on the surface. However, following treatment with 100 mg/kg AOE, the kidneys returned to a healthy reddish tone and smooth surface (Figure 3D). These data demonstrate that AOE confers renal protection in PO/Ad-induced HUA mice, with greater efficacy observed at the higher dosage level.

### 3.5 AOE reduces renal inflammation in HUA mice

**Figure 4:**
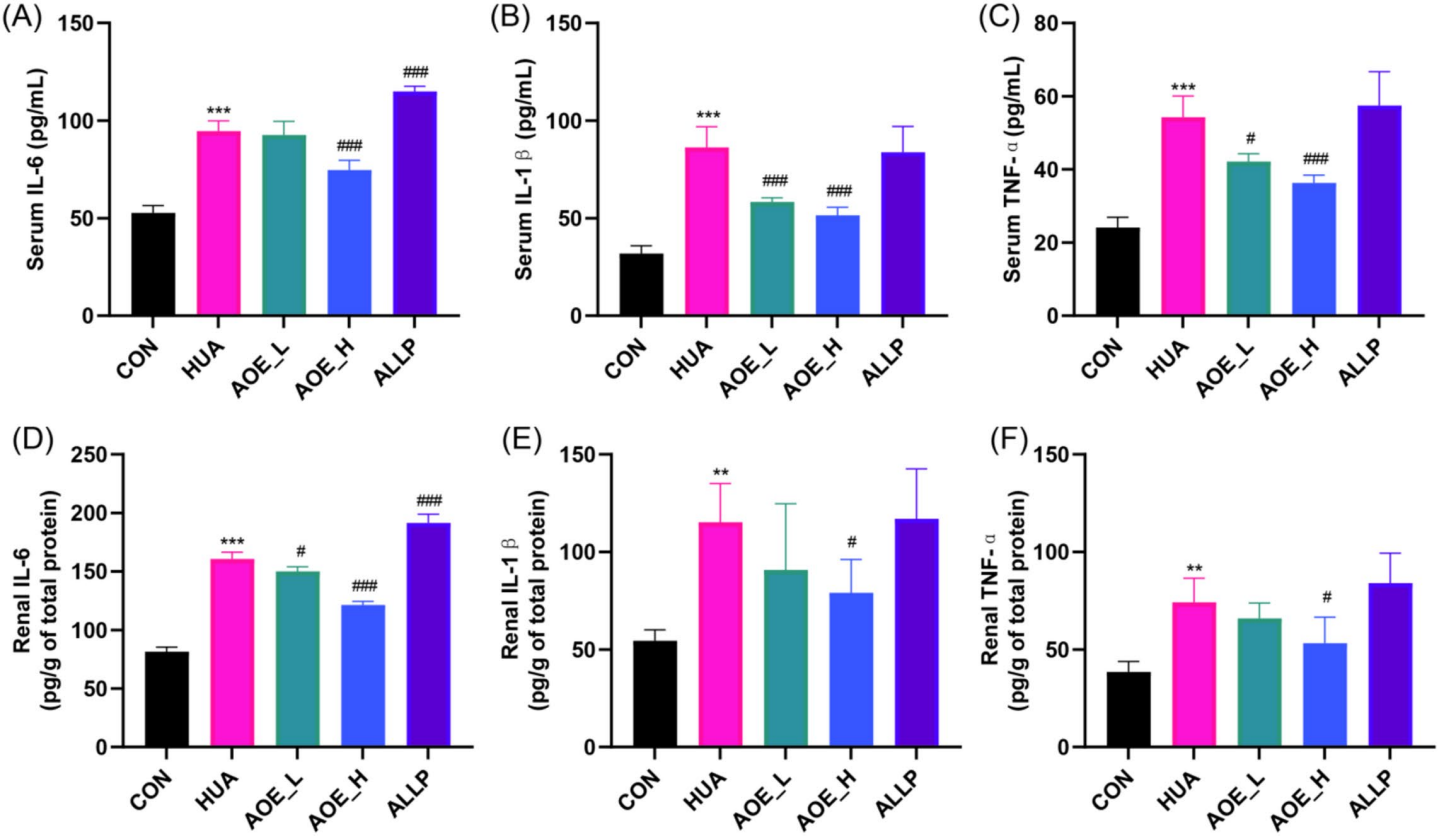
AOE ameliorated the inflammatory response in HUA mice. Serum **(A)** IL-6, **(B)** IL-1β, and **(C)** TNF-α concentrations, and the **(D)** IL-6, **(E)** IL-1β, and **(F)** TNF-α concentrations in kidney tissue. Values are expressed as mean ± standard deviation (SD). ^∗^*p* < 0.05, ^∗∗^*p* < 0.01, ^∗∗∗^*p* < 0.001 *vs* the CON group, and ^#^*p* < 0.05, ^##^*p* < 0.01, ^###^*p* < 0.001 *vs* the HUA group.

Under hyperuricemic conditions, renal tissue cells (epithelial cells, mesangial cells, endothelial cells, and tubular cells) release IL-6, IL-1β, and TNF-*α*, which promote immune cell infiltration, exacerbating renal injury. In the HUA group, IL-6, IL-1β, and TNF-α levels in serum were significantly higher than in control animals (*p* < 0.001, Figures 4A–C). AOE administration led to a marked decline in cytokine levels, with the most substantial reductions observed in the high-dose group. In kidney tissue, IL-6, IL-1β, and TNF-α were significantly upregulated in HUA mice (*p* < 0.01, Figures 4D–F), but these increases were substantially attenuated in the AOE_H group (*p* < 0.05). Allopurinol did not restore IL-1β and TNF-α expression in the kidney (*p* > 0.05 vs. HUA-treated animals), and even appeared to increase IL-6 levels. These findings further demonstrate that AOE effectively inhibits renal inflammation and contributes to the structural recovery of kidney tissue in HUA mice.

### 3.6 AOE modulates redox balance in HUA mice

**Figure 5:**
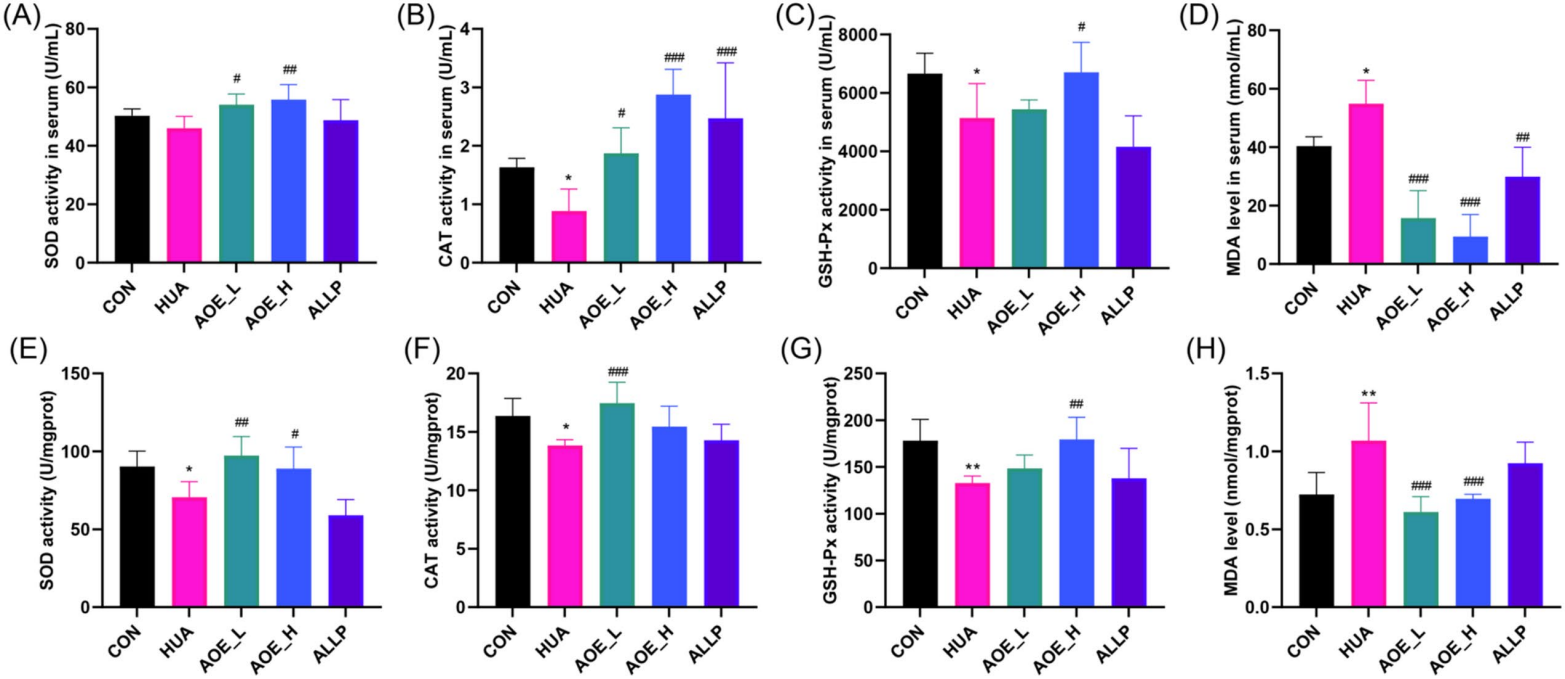
AOE ameliorated oxidative stress in HUA mice. Serum **(A)** SOD, **(B)** CAT, and **(C)** GSH-Px activity; **(D)** Serum MDA level; The SOD **(E)**, CAT **(F)**, and GSH-Px **(G)** activity in kidney tissue; **(H)** MDA level in kidney tissue. Values are expressed as mean ± standard deviation (SD). ^∗^*p* < 0.05, ^∗∗^*p* < 0.01, ^∗∗∗^*p* < 0.001 *vs* the CON group, and ^#^*p* < 0.05, ^##^*p* < 0.01, ^###^*p* < 0.001 *vs* the HUA group.

Elevated UA disrupts the redox equilibrium in renal tissues, leading to oxidative stress within the kidneys (32). To investigate the antioxidant effects of AOE, the activities SOD, CAT, and GSH-Px along with malondialdehyde (MDA) levels were measured in HUA mice. Serum from the HUA group showed reduced activities of these enzymes, while MDA concentrations were significantly higher in HUA mice than in controls (*p* < 0.05) (Figures 5A−D). A similar pattern was observed in kidney tissues, where antioxidant enzyme activities decreased and MDA levels were elevated in HUA mice (Figures 5E−H), signifying that HUA induces oxidative stress *in vivo*.

Antioxidant enzyme activities and MDA levels in kidney tissues consistently showed the same trend. These results indicate that after AOE intervention, the decline in activities in renal CAT, SOD, and GSH-Px caused by HUA was restored. Concurrently, the increase in MDA level was also reversed by AOE, as shown by 77.4 and 35.2% reduction in serum (Figure 5D) and kidney tissue (Figure 5H), respectively, post 50 mg/kg AOE treatment. By contrast, treatment with allopurinol had no significant effect on CAT, SOD, or GSH-Px activities, nor MDA levels in renal tissue from hyperuricemic mice (*p* > 0.05).

### 3.7 Transcriptome analysis

#### 3.7.1 Transcriptome sequencing and assembly

Furthermore, transcriptome sequencing (RNA-seq) was conducted from control, HUA, and AOE_H mice to investigate the molecular basis underlying the protective effects of AOE against HUA-induced renal injury. After filtering the adaptor sequences and low-quality sequences, 87.73 GB of clean data was obtained. Base quality scores over 30 ranged from 99.28 to 99.37%, indicating sufficient read quality (Supplementary Table S1). To assess the reliability of the experiment, the correlation coefficient between samples was calculated (Supplementary Figure S1). PCoA showed that control and HUA-treated animals exhibited distinct clustering and significant separation, indicating substantial alterations in gene expression between HUA and control mice. However, the genetic profile of the AOE_H group tended to converge with that of the control group (Supplementary Figure S2).

#### 3.7.2 DEG screening

**Figure 6:**
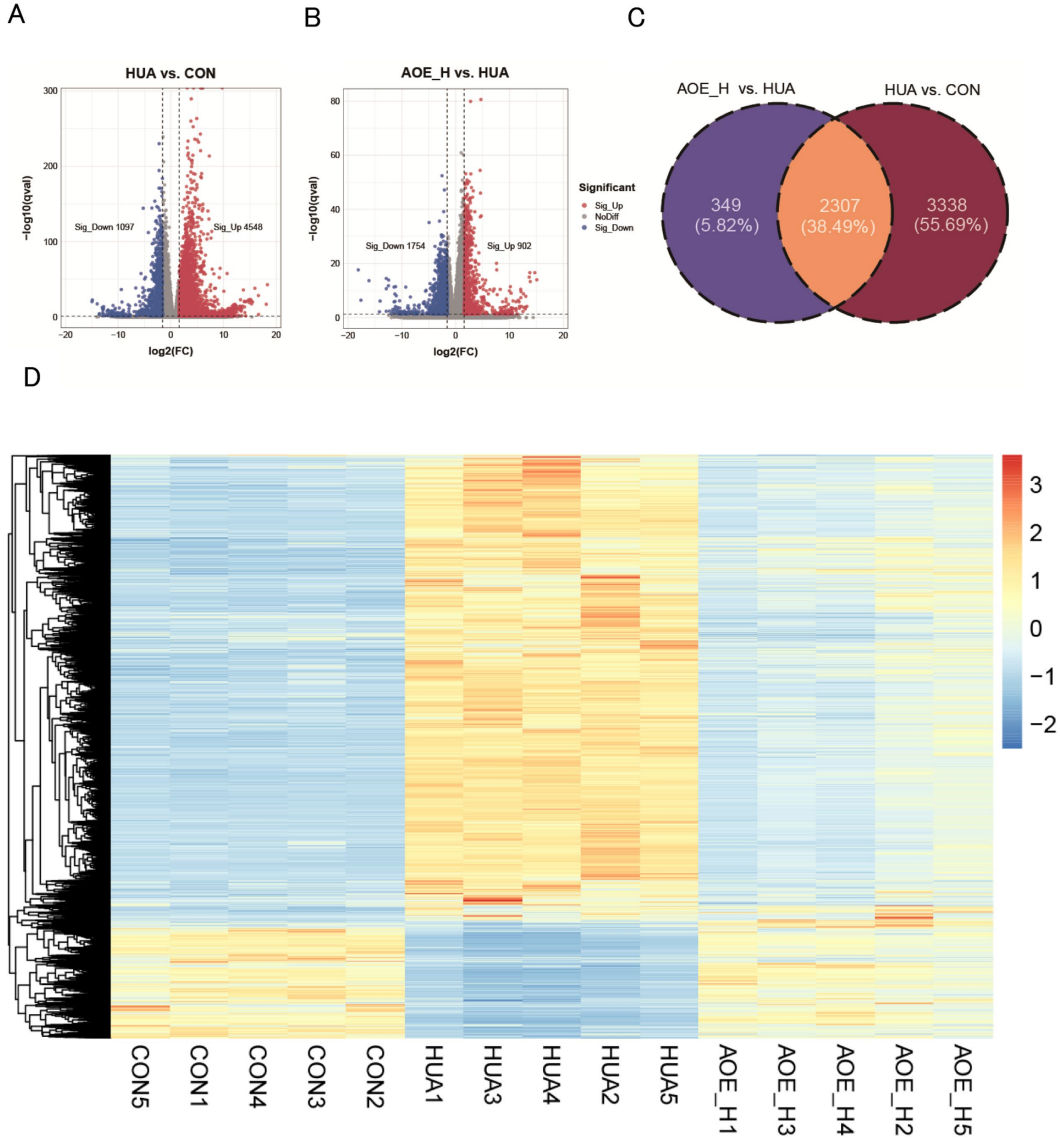
AOE treatment restored the transcript profiling in HUA mice. **(A)** Volcano plot of the DEGs between HUA and CON; **(B)** Volcano plot of the DEGs between AOE_H and HUA; **(C)** Venn diagram of gene expression between HUA vs. CON, and AOE_H vs. HUA; **(D)** The hierarchical clustering heatmap of overlapped DEGs in HUA vs. CON and AOE_H vs. HUA groups.

Using thresholds of log_2_FC over 1.5 and *p*-value below 0.05, 5,645 DEGs were identified between the HUA and control groups, comprising 4,548 transcripts with increased expression, and 1,097 with decreased expression (Figure 6A; Supplementary Table S3). In the AOE_H versus HUA comparison, 902 transcripts displayed elevated expression levels, while 1,754 showed reduced expression (Figure 6B; Supplementary Table S4). A total of 2,307 genes were common between the HUA vs. CON and AOE_H vs. HUA comparisons based on Venn analysis, accounting for 38.49% of the total (Figure 6C). Hierarchical clustering of these shared genes revealed that the transcriptional profile in HUA animals was opposite to control mice. This dysregulated pattern was normalized following AOE treatment (Figure 6D), indicating that AOE restored the disrupted gene expression profile caused by HUA.

#### 3.7.3 Enrichment analysis

**Figure 7:**
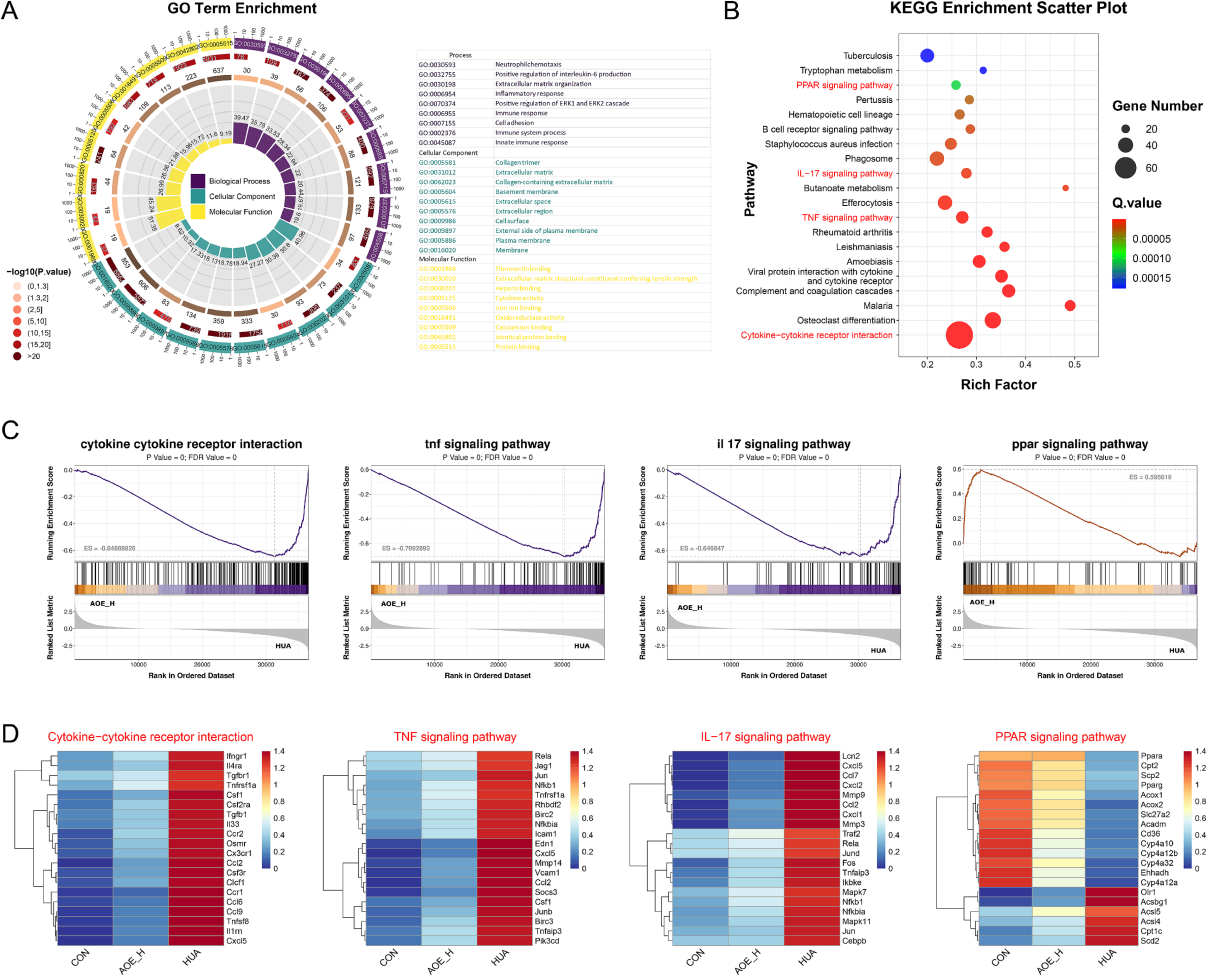
GO **(A)** and KEGG **(B)** pathway enrichment of DEGs; **(C)** Gene set enrichment analysis (GSEA) of cytokine−cytokine receptor interaction, TNF signaling pathway, IL-17 signaling pathway, and PPAR signaling pathway between AOE_H vs. HUA group; **(D)** Heatmap of the relevant gene expression from cytokine−cytokine receptor interaction, TNF signaling pathway, IL-17 signaling pathway, and PPAR signaling pathway.

To further explore the functional roles of the shared DEGs, enrichment analysis was carried out using the GO and KEGG databases. Numerous signaling pathways involved in inflammatory processes and redox imbalance were significantly enriched. GO analysis revealed significant involvement in biological processes, including interleukin-6 regulation, inflammatory response, immune activation, and oxidoreductase function (Figure 7A). KEGG pathway analysis further indicated strong associations with immune-related cascades, including cytokine**-**cytokine receptor interactions, TNF and IL-17 signaling, and the PPAR pathway (Figure 7B). GSEA demonstrated that AOE administration suppressed the activation of pro-inflammatory pathways in HUA mice, while simultaneously restoring activity within the PPAR pathway (Figure 7C). These results suggest that the therapeutic efficacy of AOE in HUA-induced renal impairment is primarily mediated through the modulation of inflammatory and oxidative stress mechanisms.

Surprisingly, the expression of genes related to cytokine-cytokine receptor interactions and inflammatory signaling cascades, such as *CCL2*, *Cxcl5*, *MMP9*, *Nfkb1*, and *RelA*, was markedly increased in HUA mice (Figure 7D). By contrast, AOE administration reduced inflammatory marker expression, suggesting that AOE mitigates renal injury primarily through the modulation of inflammation-associated pathways. Moreover, the down-regulated *Pparα*, *Cpt2*, and the *Pparg* genes in the PPAR signaling pathway of HUA mice were restored by AOE treatment.

### 3.8 RT-qPCR validation of DEGs

**Figure 8:**
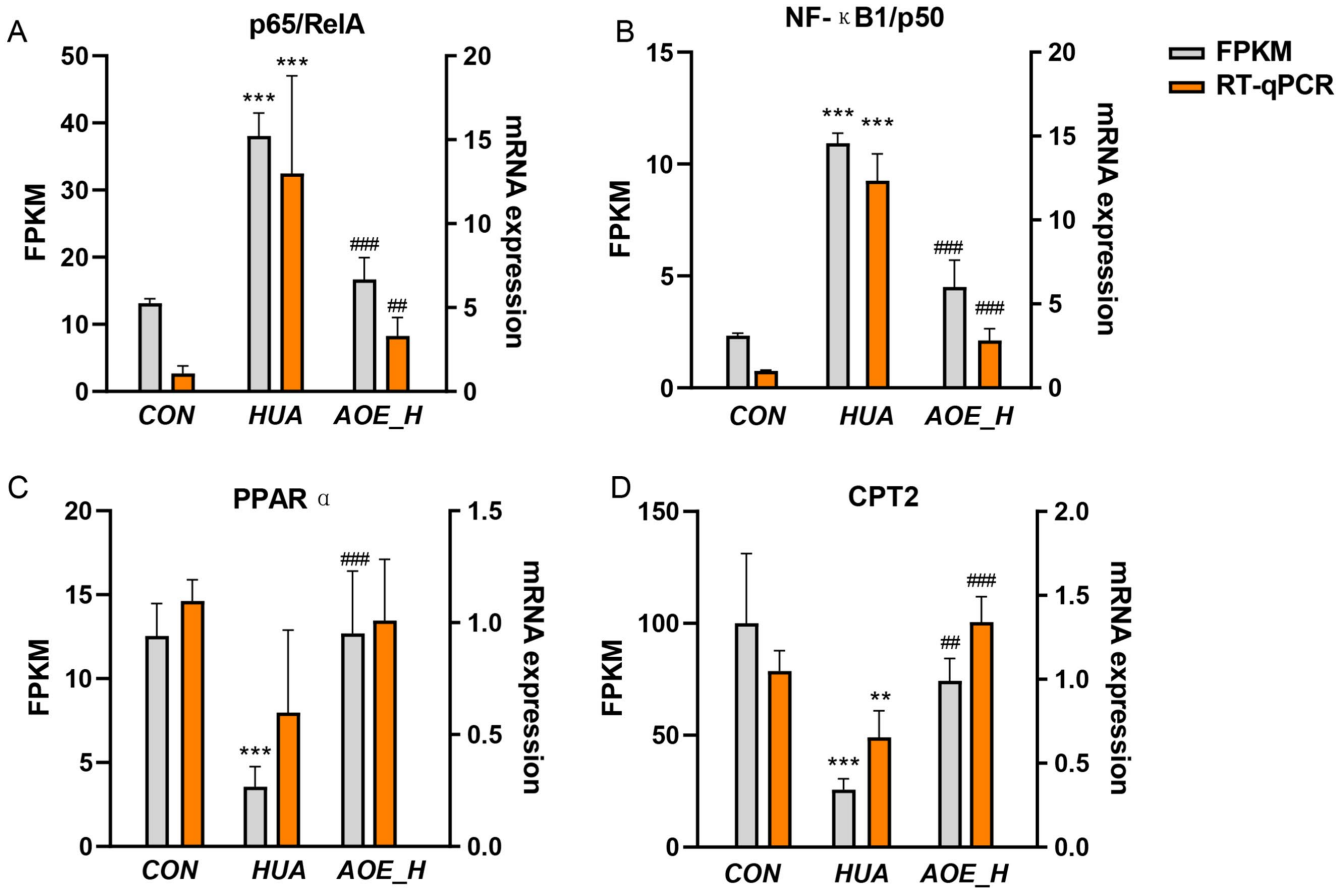
The transcriptome-revealed FPKM values of the DEGs. **(A)** p65/RelA; **(B)** NF-κB1/p50; **(C)** *PPARα*; **(D)** *Cpt2*. Values are expressed as mean ± standard deviation (SD). ^∗^*p* < 0.05, ^∗∗^*p* < 0.01, ^∗∗∗^*p* < 0.001 *vs* the CON group, and ^#^*p* < 0.05, ^##^*p* < 0.01, ^###^*p* < 0.001 *vs* the HUA group.

To confirm the transcriptomic findings, representative DEGs associated with TNF signaling pathway, IL-17 signaling pathway, and the PPAR pathway were chosen for validation by RT-qPCR. Gene expression profiles obtained from RT-qPCR fully mirrored the trends from the RNA-seq data (Figure 8). This alignment between the two datasets reinforces transcriptomic data robustness and reliability.

## 4 Discussion

Chronic HUA levels typically result in renal function damage because the kidneys struggle to filter out excess UA. This promotes the formation of urate crystals (33), which can adhere to kidney tubules, triggering inflammation and subsequent renal injury (34), potentially causing HN (10). On the other hand, elevated uric acid levels contribute to the overproduction of reactive oxygen species, leading to cellular oxidative imbalance and tissue injury (9). Increasing evidence shows that inflammatory processes and oxidative stress are central drivers in the pathological transition from HUA to HN (35). In this context, natural, plant-derived bioactive compounds with antioxidant and anti-inflammatory properties have attracted increasing attention as alternatives for managing HUA while protecting against renal injury (9, 13). Aloe-emodin (AOE), the primary anthraquinone constituent derived from aloe and rhubarb, contains antioxidant, anti-inflammatory, antibacterial, and hepatoprotective properties (24, 36). This suggests that AOE has the potential to be a natural candidate against HUA and HUA-induced kidney injury.

The PO/Ad-induced mouse model reliably replicates HUA-associated renal dysfunction, making it an indispensable method for researchers aiming to conduct in-depth investigations into the pathophysiology of HN (37). Our results show that AOE decreases serum UA levels in the PO/Ad-induced HUA mouse model. Our data further demonstrate that AOE treatment suppressed the activities of XOD and ADA elevated by PO/Ad exposure. Similarly, Meng et al. reported that rhubarb acid, an anthraquinone compound derived from *Rheum palmatum*, also exhibited a UA-lowering effect by inhibiting XOD activity (post 70–300 mg/kg/d) (19). Notably, XOD and ADA are critical enzymes in the liver that significantly influence UA synthesis, with XOD serving as a major regulator of systemic uric acid production (38, 39). Allopurinol, an XOD inhibitor, is among the most effective clinical treatments for HUA (40), but is therapeutically-restrictive as it may produce liver and kidney toxicity (11, 41). In our investigation, evaluation of systemic toxicity through measurements of body weight and organ indices, specifically for the kidneys and liver, demonstrated that AOE exhibited favorable safety characteristics while also alleviating hyperuricemia and exerting protective effects on organ health.

A growing body of research increasingly supports a close association between HUA and renal damage. Elevated levels of BUN and serum creatinine are key indicators of impaired renal function (26). Renal injury decreases the clearance of urea and creatinine, thus as evidenced by increasing levels of BUN and serum Cr (42). This study demonstrated that treatment with AOE effectively reduced BUN and serum Cr levels, indicating its kidney-protective effect in HUA mice. Furthermore, the histopathological analysis using H&E staining confirmed that HUA status led to kidney tissue deterioration. However, the administration of AOE resulted in a remarkable transformation. The intervention was observed to restore the integrity of renal tissue structure, significantly reducing the pathological features indicative of renal distress. These results suggest that AOE was nephroprotective in PO/Ad-induced hyperuricemic mice.

Excessive UA levels in the body trigger the innate immune system, leading to a cascade of adverse effects on renal cell morphology and function. In general, this process begins with immune system activation, which subsequently triggers the secretion of inflammatory mediators (43). In our study, PO/Ad exposure induced an inflammatory response in HUA mice, as evidenced by increasing serum and renal proinflammatory cytokines levels. AOE significantly suppressed high proinflammatory cytokine levels, ameliorating inflammatory responses in HUA mice (Figure 4). Furthermore, mounting evidence suggests that oxidative injury, driven by elevated uric acid levels, is a key contributor to renal cellular damage and inflammation (44). Under normal conditions, maintaining an appropriate level of UA is conducive to eliminating reactive oxygen species (45). However, elevated blood UA levels increase oxidase activity and decrease SOD function, leading to oxidative tissue damage (46, 47). As demonstrated in this study, oxidative injury was noticed in the kidneys of PO/Ad-induced HUA mice (Figure 5). Similar studies have consistently reported significant oxidative damage to HUA mice, characterized by increased MDA accumulation and diminished activity of key antioxidant enzymes (9). Therefore, targeting oxidative stress pathways has emerged as a promising strategy for mitigating HUA-associated kidney injury (47). For example, Qian et al. demonstrated that linarin (dose of 120 mg/kg/d), the flavonoids compounds in *Chrysanthemum indicum* L., could alleviate oxidative stress of HUA mice, thereby delaying the progression of HN (9). Chen et al. demonstrated that the flavonoid extracts from saffron by-product could effectively improve the symptom of kidney injury in HUA rat via enhancing antioxidant capacity (48). In this study, AOE treatment (post 50–100 mg/kg/d) significantly reversed the increased MDA levels and the decreased antioxidant levels in HUA mice, suggesting a regulatory effect on oxidative damage. In summary, our results provide compelling evidence that AOE confers renal protection in the PO/Ad-induced HUA mouse model by reducing inflammation and oxidative stress.

Our transcriptome RNA sequence results also show that the molecular mechanisms of AOE against HUA-induced renal injury linked to inflammatory responses and oxidative stress. Key regulatory pathways implicated in these molecular events include the IL-17 axis, TNF signaling, and PPAR pathways. Among them, the TNF cascade plays a critical role in modulating immune activity and orchestrating inflammatory processes (49, 50). Furthermore, IL-17, a cytokine predominantly released by Th17 cells, increases NF-κB signaling activity and promotes the production of pro-inflammatory mediators within renal epithelial and endothelial tissues (26). Importantly, hyperuricemic conditions were associated with upregulation of the *RelA* and *Nfkb1* genes, core elements of the TNF and IL-17 signaling networks. Treatment with AOE effectively normalized this aberrant gene expression. *RelA* and *Nfkb1* are important members of the NF-κB family that regulate a broad spectrum of biological functions, encompassing immune surveillance, inflammatory regulation, and programmed cell death (51). Typically, the NF-κB complex consists of a heterodimer composed of p65 (*RelA*) and p50 (*Nfkb1*) subunits (52). Once activated, this dimer drives the transcription of pro-inflammatory effectors, perpetuating cellular inflammation (53). Our transcriptomic analyses revealed that AOE substantially suppressed *RelA* and *Nfkb1* expression, a finding further validated by RT-qPCR, which showed significant declines in p65/RelA and NF-κB1/p50 transcript levels following AOE administration in HUA mice.

**Figure 9:**
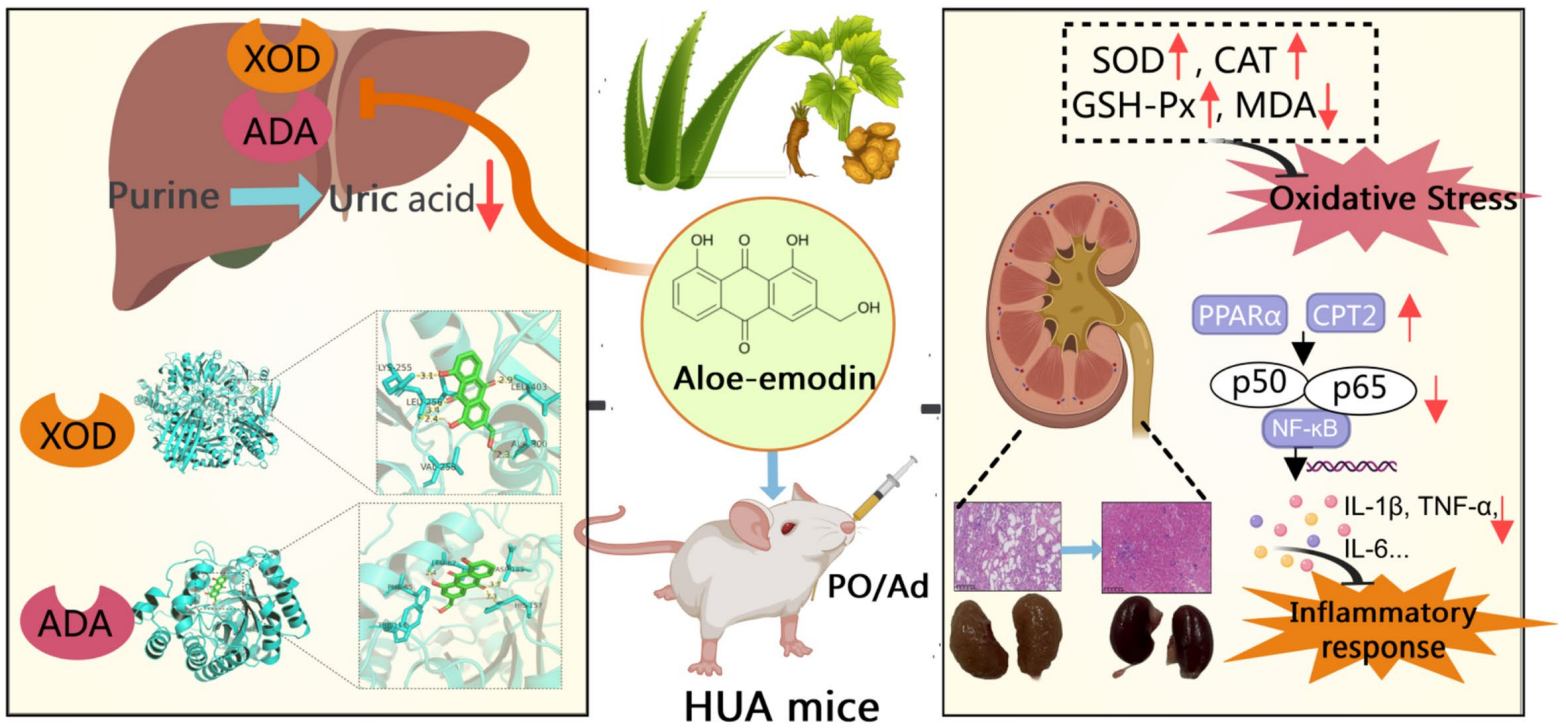
The potential mechanism underlying the protective effects of AOE against hyperuricemia and renal injury.

Biological activities within cells are often governed by coordinated networks of numerous interacting genes that function in concert to regulate cellular processes. Natural phytochemicals, particularly those derived from medicinal plants, are recognized for their ability to modulate multiple molecular targets and signaling pathways concurrently. For example, quercetin mitigates renal inflammation by reducing TNF-*α* and IL-1β levels through reactive oxygen species (ROS)-dependent mitogen-activated protein kinase (MAPK) and NF-κB signaling (39). In this study, AOE demonstrated multi-targeted therapeutic potential in ameliorating kidney damage induced by HUA (Figure 9). Beyond its influence on IL-17 and TNF-associated pathways, AOE also regulated genes involved in the PPAR axis in HUA mice. Transcriptomics data revealed increased expression of key PPAR pathway components, including *Pparα*, *Cpt2*, and *Pparg* in AOE-treated mice. The PPAR system coordinates lipid metabolism, attenuates oxidative stress, and modulates immune responses (54). Activation of PPARα and PPARγ improves oxidative stress and inhibits the Th17-mediated inflammatory response (55, 56). RT-qPCR confirmed that AOE increased *PPARα*, *Cpt2*, and *PPARγ* mRNA levels in kidney tissue from HUA mice. Furthermore, PPARγ activation negatively regulates NF-κB p65 activity, thereby dampening pro-inflammatory cytokine production and improving tissue inflammation (54). Our results consistently showed that AOE treatment upregulated PPARγ transcription while concurrently downregulating p65/RelA and NF-κB1/p50 mRNA expression in HUA mice, suggesting that its renoprotective actions may be mediated through modulation of the PPAR/NF-κB signaling axis. However, there are some limitations of this study that need to be addressed. First, this study conducted preclinical study using HUA mouse model to investigate the possible efficacy of AOE in the treatment of HUA-induced renal injury. Due to the significant interspecies differences between rodents and humans, the translational value of preclinical models is limited (18). Therefore, more experimental studies, such as clinical data, pharmacokinetics, toxicity, and safety data are needed in the future. Besides, although RT-qPCR confirmed that AOE increased mRNA expression of related genes involving PPAR and NF-κB pathways, further protein-level evidence (e.g., western blotting or immunohistochemistry) should be conduct to validate the proposed molecular mechanisms. Moreover, further functional gene validation (e.g., PPAR/NF-κB inhibitors or the gene knockdown) are needed to confirm our results in the future.

## 5 Conclusion

In the current study, we conducted a preclinical study to assess the efficacy of AOE in treating HUA and its associated renal injury. AOE administration suppressed XOD and ADA activity in HUA mice, reduced serum uric acid concentrations, improved inflammation and oxidative stress, and alleviated the HUA-induced renal injury. Furthermore, AOE conferred substantial nephroprotective effects in HUA mice, mainly through its regulatory influence on oxidative imbalance and inflammation targeting PPAR/NF-κB signaling axis. In summary, our results indicate that AOE is a promising plant-derived therapeutic with a favorable safety profile for managing HUA and its associated renal complications. However, in-depth investigations and further studies are needed in the future to validate the application of AOE in the treatment of HUA and its associated renal injury.

## Data Availability

The datasets presented in this study can be found in online repositories. The names of the repository/repositories and accession number(s) can be found here: https://www.ncbi.nlm.nih.gov/bioproject/PRJNA1299542.

## Footnotes

^1^ https://www.rcsb.org/

^2^ https://github.com/OpenGene/fastp

^3^ http://bioinf.wehi.edu.au/featureCounts/

^4^ http://deweylab.github.io/RSEM

^5^ http://www.geneontology.org/

^6^ https://www.omicstudio.cn/tool
