# Supplementary material for: Aloe-emodin attenuates hyperuricemia-induced renal injury in mice by mitigating inflammation and oxidative stress
Source: Front Nutr. 2025 Oct 15;12:1677560. doi: 10.3389/fnut.2025.1677560 (PMC12568037; doi:10.3389/fnut.2025.1677560)
Supplement: Supplementary file 1 [file Table_1.doc]

## Supplementary materials

## Aloe-emodin attenuates hyperuricemia and renal injury in mice by mitigating the inflammatory response and oxidative stress

Shengfeng Wang^a,1^, Quanfeng Zhu^b,1^, Chengcheng Zhang^c^, Shuang Hu^d^, Daqun Liu^c^, Jiawen Yu^b^, Xiao Liu^b^, Yan Chen^a,*^, Guojun Jiang^b,*^

^a^ Laboratory of Animal Center, Research Center of Analysis and Measurement, Zhejiang University of Technology, Hangzhou 310014, PR China

^b^ Affiliated Xiaoshan Hospital, Hangzhou Normal University, Hangzhou, 311201

^c^ Food Science Institute, Zhejiang Academy of Agricultural Sciences, Hangzhou 310021

^d^ School of Pharmacy, Hangzhou Normal University, Hangzhou, 311121, China

**Chemicals and reagents**

Potassium oxonate (PO, S17112) were acquired from Yuanye Bio-Technology Co., Ltd (Shanghai, China). Adenine (Ad, A108805) and [allopurinol](https://www.sigmaaldrich.cn/CN/en/substance/allopurinol13611315300) (ALLP, A8003) were purchased from Aladdin (Shanghai, China) and Sigma-Aldrich (St. Louis, USA), respectively. Carboxymethyl cellulose (CMC, IS9000) was purchased from Solarbio (Beijing, China). Assay kits for XOD (A002-1-1), ADA (A048-2-1), malondialdehyde (MDA, A003-1-2), superoxide dismutase (SOD, A001-3-2), glutathione peroxidase (GSH-Px, A005-1-2), and catalase (CAT, A007-1-1) were obtained by Nanjing Jiancheng Bioengineering Institute (Jiangsu, China). The enzyme-linked immunosorbent assay (ELISA) kits for interleukin (IL)-6, IL-1β, and tumor necrosis factor alpha (TNF-α) were obtained by Shanghai Enzyme-linked Biotechnology Co., Ltd. (Shanghai, China).

Table S1. PCR primer sequence table

| Gene | Primer sequence (forward, reverse) |
| --- | --- |
| NF-κB p65 (mouse) | AGGCTTCTGGGCCTTATGTG |
|  | TGCTTCTCTCGCCAGGAATAC |
| NF-κB1 (p50) (mouse) | ATGGCAGACGATGATCCCTAC |
|  | TGTTGACAGTGGTATTTCTGGTG |
| PPARα (mouse) | AGAGCCCCATCTGTCCTCTC |
|  | ACTGGTAGTCTGCAAAACCAAA |
| PPARγ (mouse) | TCGCTGATGCACTGCCTATG |
|  | GAGAGGTCCACAGAGCTGATT |
| CPT-2 (mouse) | CAGCACAGCATCGTACCCA |
|  | TCCCAATGCCGTTCTCAAAAT |
| GAPDH (mouse) | TGTTCCAGTATGACTCCACTCA |
|  | CACCAGTAGACTCCACGACAT |

Table. S2. Quality analysis of transcriptome sequencing data of different samples.

| Sample | Raw reads | Clean reads | Clean bases | Q30% | GC content% |
| --- | --- | --- | --- | --- | --- |
| CON_1 | 40128820 | 38815800 | 5.82G | 99.31 | 48.50 |
| CON_2 | 39323110 | 38014178 | 5.70G | 99.35 | 48.50 |
| CON_3 | 36864478 | 35685404 | 5.35G | 99.33 | 48 |
| CON_4 | 38823342 | 37615440 | 5.64G | 99.36 | 48.50 |
| CON_5 | 41523700 | 40240856 | 6.04G | 99.32 | 48.50 |
| HUA_1 | 38247734 | 36983314 | 5.55G | 99.32 | 49.50 |
| HUA_2 | 39955186 | 38499950 | 5.77G | 99.30 | 49.50 |
| HUA_3 | 40033070 | 38540454 | 5.78G | 99.28 | 49.50 |
| HUA_4 | 35236110 | 33969254 | 5.10G | 99.34 | 49.50 |
| HUA_5 | 35361570 | 34097836 | 5.11G | 99.28 | 49.50 |
| AOE_H_1 | 51516514 | 49794236 | 7.47G | 99.36 | 48 |
| AOE_H_2 | 42585054 | 41133568 | 6.17G | 99.33 | 48.50 |
| AOE_H_3 | 41844136 | 40526644 | 6.08G | 99.35 | 48.50 |
| AOE_H_4 | 41260822 | 39922676 | 5.99G | 99.34 | 48.50 |
| AOE_H_5 | 42526700 | 41055818 | 6.16G | 99.30 | 48.50 |


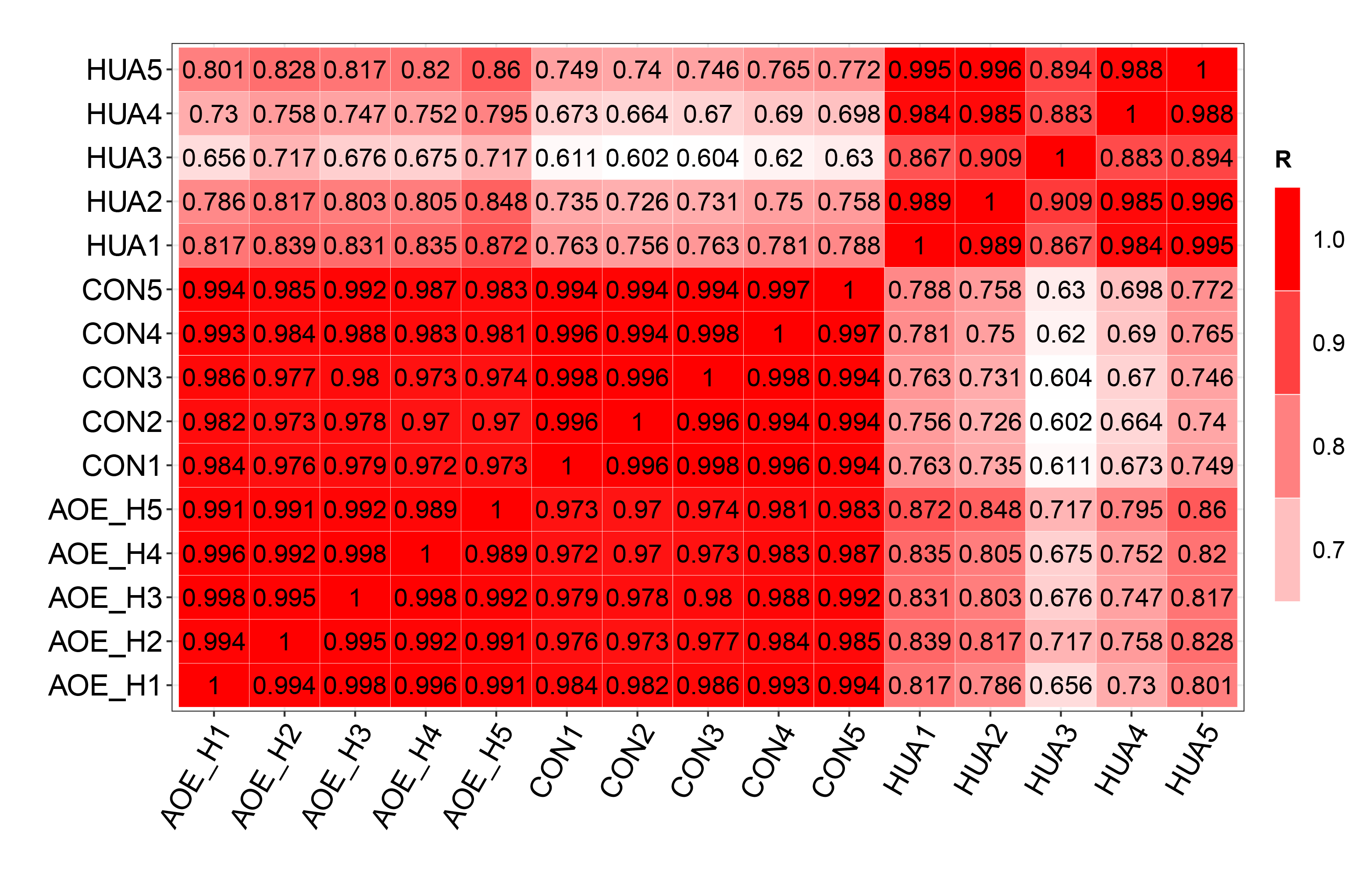


**Figure S1.** Correlation thermograms between samples in different groups.


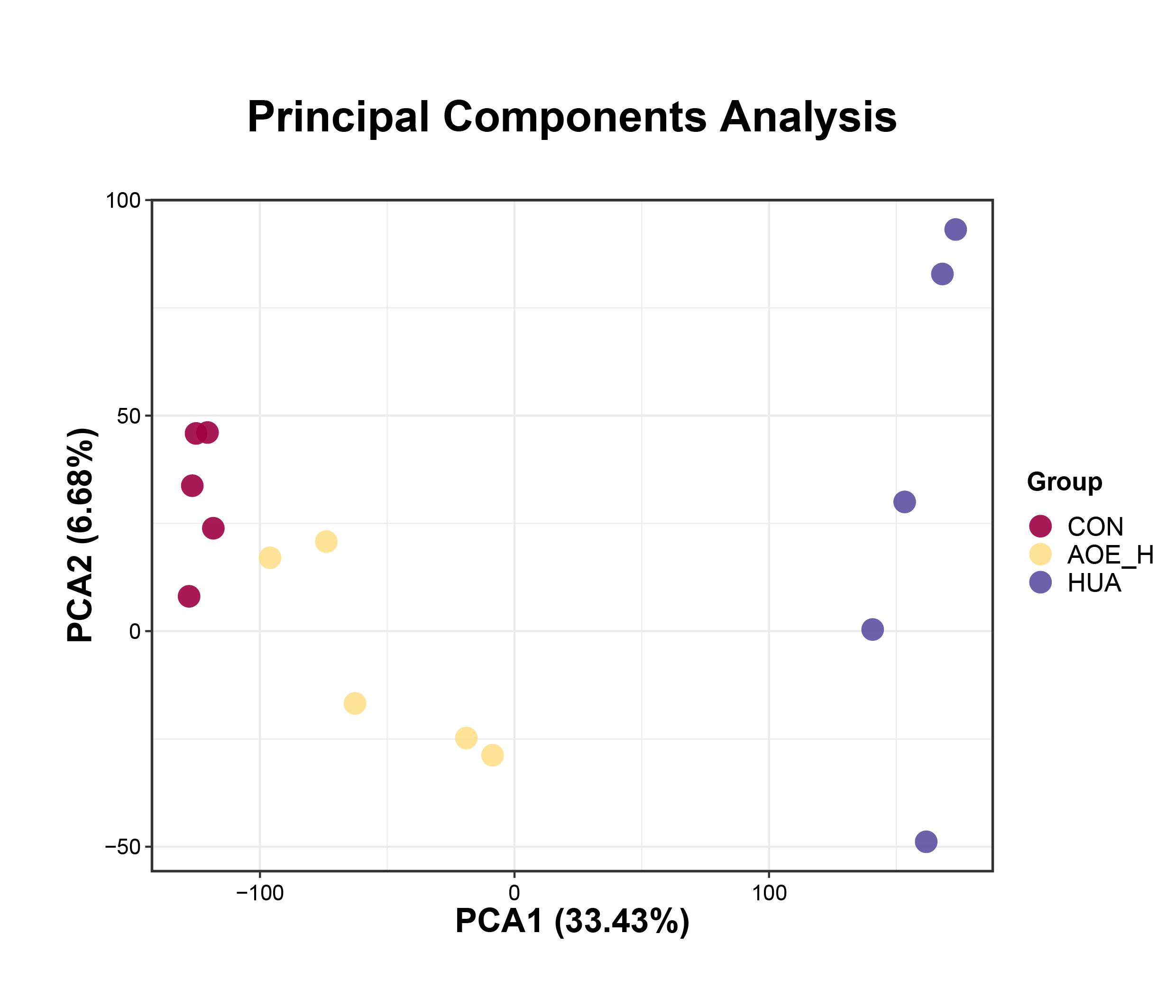


**Figure S2.** Principal component analysis of the differences in gene expression profiles.
